# Design of Beam Shaping Assemblies for Accelerator-Based BNCT With Multi-Terminals

**DOI:** 10.3389/fpubh.2021.642561

**Published:** 2021-03-11

**Authors:** Guangru Li, Wei Jiang, Lu Zhang, Weiqiang Chen, Qiang Li

**Affiliations:** ^1^Institute of Modern Physics, Chinese Academy of Sciences, Lanzhou, China; ^2^College of Life Sciences, University of Chinese Academy of Sciences, Beijing, China; ^3^Key Laboratory of Heavy Ion Beam Radiation Biomedicine, Chinese Academy of Sciences, Lanzhou, China; ^4^Gansu Provincial Key Laboratory of Heavy Ion Beam Radiation Medical Application, Lanzhou, China

**Keywords:** accelerator-based BNCT, beam shaping assembly, thermal neutron, epithermal neutron, multi-terminal

## Abstract

To moderate fast neutrons produced by accelerator to appropriate therapeutic energies for boron neutron capture therapy (BNCT), beam shaping assembly (BSA) is required definitely. In this work, based on a model of 2.5 MeV/30 mA proton accelerator, the Monte Carlo simulation software MCNPX was employed to design multi-terminal BSAs. All parameters for both the thermal and epithermal neutron beams at the exit ports of the designed BSAs meet the treatment recommendation values proposed by the International Atomic Energy Agency (IAEA). The clinical parameters of the thermal and epithermal neutron beams were also calculated for clinical indication consideration.

## Introduction

Boron neutron capture therapy (BNCT) is a novel modality of radiation cancer therapy. Boron compounds are administered to tumor cells, then the tumor is irradiated with neutrons, inducing the ^10^B (n, α)^7^Li nuclear reaction:

(1) $$\begin{array}{r} {\;}^{10}\text{B}+{\text{n}}_{\text{th}}\to^{11}\text{B}\to^{4}\text{He}\left(1.78\text{ MeV}\right){+}^{7}\text{Li}\left(1.01\text{ MeV}\right)\;\left(6.3\text{\%}\right)\\ {\;}^{10}\text{B}+{\text{n}}_{\text{th}}\to^{11}\text{B}\to^{4}\text{He}\left(1.47\text{ MeV}\right){+}^{7}\text{Li}\left(0.84\text{ MeV}\right)+\gamma \left(0.48\text{ MeV}\right)\;\left(93.7\text{\%}\right) \end{array}$$

Both α particles and ^7^Li nuclei deposit their energies along their very short paths, which are comparable to the size of cells. As a result, tumor cells are destroyed accurately without harming healthy tissues (1).

BNCT was firstly proposed by G. Locher in 1936 (2) and firstly practiced by W. Sweet in 1951 for the clinical trial of glioma (3). Over the past two decades, many research groups around the world have continued the work of W. Sweet and the others, particularly the pioneering clinical work of Hatanaka (4). Subsequently, clinical trials of BNCT were conducted in the United States, Sweden, Finland, the Czech Republic, Argentina, the European Union (centered on Finland) and Japan.

Nuclear reactors were firstly used to produce neutrons for BNCT. However, although they could provide high-intensity neutron beam, they have numerous shortcomings: most of them are located far from hospitals, and are also very expensive. Besides, nuclear reactors have too huge size to be suitable for being used in hospital. Accelerator-based BNCT (AB-BNCT) facilities therefore are being developed to replace nuclear reactors. In AB-BNCT, fast neutrons are obtained by bombarding lithium or beryllium target with protons. However, the fast neutrons produced by this method cannot be used for BNCT treatment directly and need to be moderated by beam shaping assembly (BSA). The functions of BSA are: (1) slow fast neutrons down to thermal neutrons (<0.5 eV) or epithermal neutrons (0.5 eV−10 keV), (2) reduce the composition of fast neutron, thermal neutron and γ ray as much as possible, and (3) collimate neutron beam. The thermal neutron is suitable for treating superficial lesions while the epithermal neutron is for treating deep ones. BSA is mainly composed of the following components: moderator, reflector, gamma filter, collimator, etc., and thermal neutron filters are also required if neutrons need to be moderated to the energy range of epithermal neutrons.

Currently, the proposed BSA designs around the world mainly focus on generating epithermal neutrons which are essential for the treatment of deep-seated tumors, such as the BSAs in Tsukuba University (5), Nagoya University (6, 7), and Kyoto University (8). However, thermal neutron beam cannot be ignored anyway. It is applicable to the treatment of superficial tumors, such as melanoma, as well as cell and animal pre-clinical experiments. Therefore, multiple BSAs were designed for the generation of thermal and epithermal neutrons, respectively, and both of them fulfill the IAEA recommended values (9) which are listed in Table 1. In this work, the Monte Carlo simulation program MCNPX was used to design multiple BSAs based on an AB-BNCT model and the clinical parameters of the thermal and epithermal neutrons generated from the multiple BSAs were calculated, aiming at providing reference for the construction of AB-BNCT facility.

**Table 1:** Neutron beam parameters and IAEA recommended values.

**Thermal neutron beam parameters**	**Recommended values**	**Epithermal neutron beam parameters**	**Recommended values**
Thermal neutron flux Φ_th_ (cm^−2^s^−1^)	≥1 × 10^9^	Epithermal neutron flux Φ_epith_ (cm^−2^s^−1^)	≥1 × 10^9^
Thermal neutron ratio Φ_th_/Φ_total_	>0.9	Thermal neutron ratio Φ_th_/Φ_epith_	≤0.05
Epithermal and fast neutron component D_epi−fast_/Φ_th_ (Gy cm^2^)	≤2 × 10^−13^	Fast neutron component D_fast_/Φ_epith_ (Gy cm^2^)	≤2 × 10^−13^
Gamma component D_γ_/Φ_th_ (Gy cm^2^)	≤2 × 10^−13^	Gamma component D_γ_/Φ_epith_ (Gy cm^2^)	≤2 × 10^−13^
J/Φ	>0.7	J/Φ	>0.7
Thermal energy group Φ_th_		E<0.5 eV	
Epithermal energy group Φ_epith_		0.5 eV≤E≤10 keV	
Fast energy group Φ_fast_		E>10 keV	

## Methods and Materials

### Initial BSA Model

**Figure 1:**
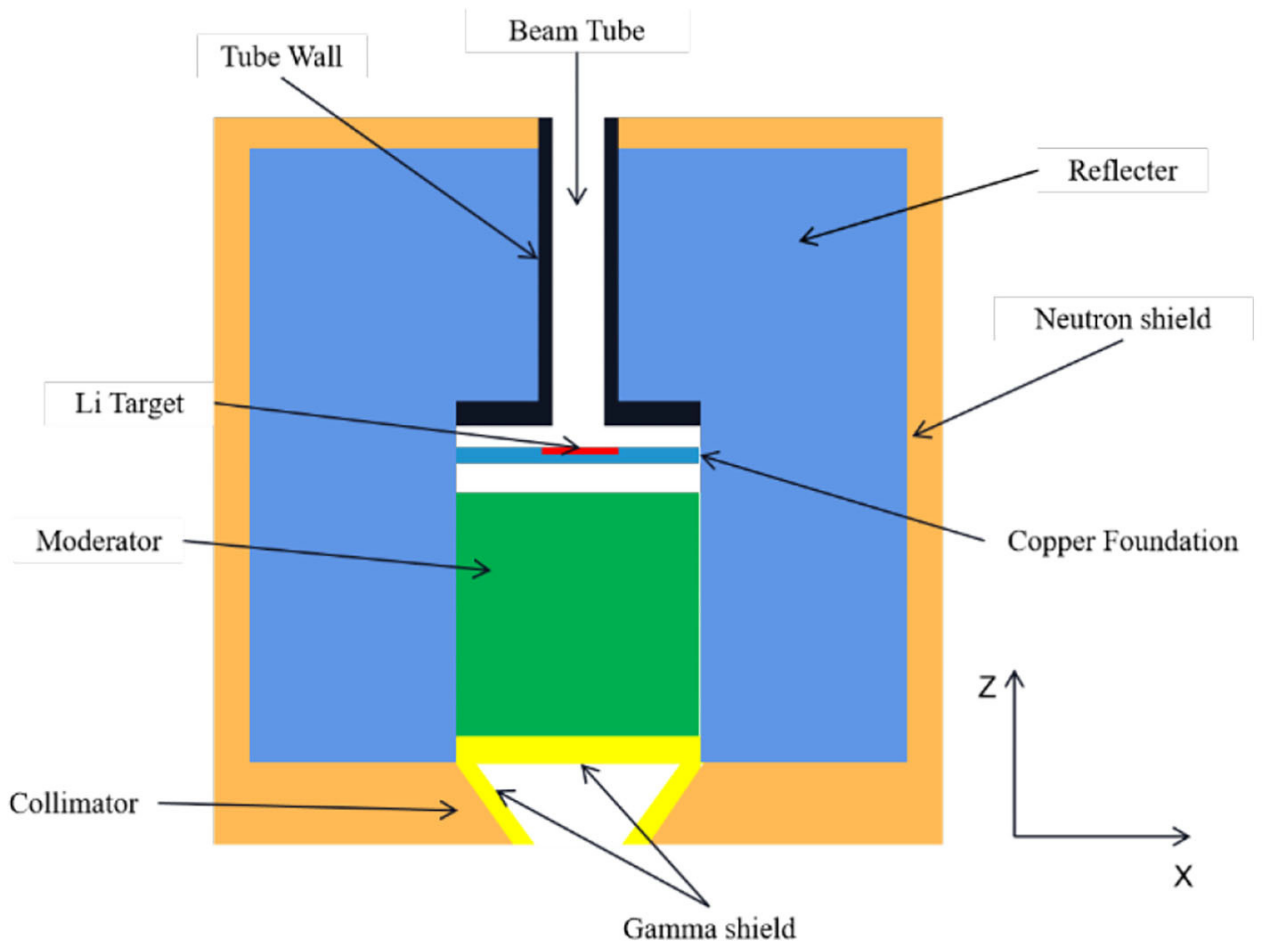
Structure of initial design of BSA.

An initial BSA model was proposed and shown in Figure 1. The whole BSA has a cylinder structure with lithium target and beam channel located in the central axis of the cylinder. The lithium target is 10 cm in diameter and 100 μm in thickness, and there is a copper holder of 2.3 cm in thickness below the target, which plays a role in heat dissipation and structure support. Thirty milliampere protons would generate lots of heat in target indeed and Li target has a low melt pointing. So, it is necessary to consider a cooling system for the Li target. In this work, copper was used to roughly represent the cooling system for target. The beam pipe is made of 316L stainless steel with a thickness of 1 cm for 2.5 MeV proton transport, and the thickness of the stainless steel above the target is 4 cm. In this way, the radiation damage of recoiling neutrons and protons can be reduced. Boron containing polyethylene (10 wt% natural B) is used in the outer side of the BSA as an absorption shield for neutrons. On the bottom of the collimator, a gamma shield is designed in the inner side of the collimator to further reduce the gamma component in the beam. The diameter of the BSA beam port is set to 14 cm. Finally, we used the MCNPX software to calculate the physical and clinical parameters of thermal and epithermal neutrons.

In addition, in the optimization process of BSA for thermal neutrons, the energy of thermal neutron is close to the kinetic energy of nuclear thermal motion in materials of reflector, moderator etc. So, the library of the thermal scattering law data S (α, β) may be required. This library plays an important role in describing the transport of thermal neutrons (10). This work used the ENDF/B-VII.0 cross section library for the simulation of thermal neutron scattering.

### Neutron Source

**Figure 2:**
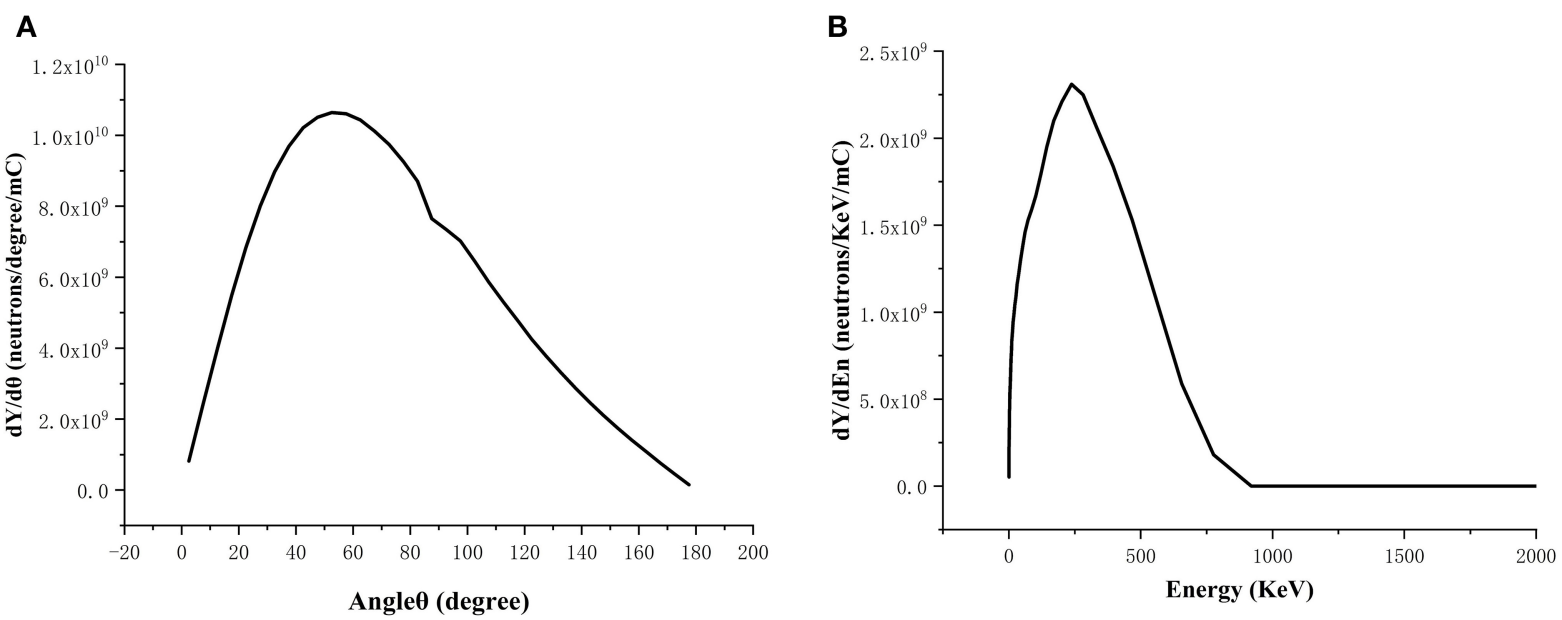
Spectrum **(A)** and angular distribution **(B)** of the neutron beam generated from bombarding Li target by 2.5 MeV/30 mA protons.

The neutron beam generated from bombarding lithium target by 2.5 MeV protons was simulated using the MCNPX software, whose spectrum and angular distribution are shown in Figure 2. The neutron beam was made as a dumb data file, which acted as a neutron source used in the BSA optimization. In this way, the computation time was greatly reduced.

### Moderator and Reflector

The most important part of BSA is moderator. Its role is to moderate the energy of neutrons produced by protons into the energy range of thermal or epithermal neutrons without producing excessive gamma rays. Thus, the moderator should have a high scattering cross section at desired energies (thermal or epithermal energy), low one for undesired energies (thermal or fast energy) and absorption cross section, avoiding loss of neutron intensity and producing large quantities of gamma-rays (11). In the optimization process, different moderator materials (Fluental, TiF_3_, CaF_2_, Al, AlF_3_ and MgF_2_ for epithermal neutron beam BSA, and D_2_O, normal polyethylene and graphite for thermal neutron BSA, respectively) were considered (12–14).

Another important part is reflector which is used to reflect scattered neutrons back into the beam. Reflector should have a low absorption cross section, a high elastic scattering cross section for thermal or epithermal neutrons and also a large mass number in which less loss of energy with elastic collision. We considered Teflon, Pb, 316L stainless steel, BeO, and Al_2_O_3_ for thermal neutron beam BSA, and Teflon, Al_2_O_3_, Pb for epithermal neutron beam BSA, respectively.

### Neutron and Gamma Filters

To minimize the damage to healthy tissue around the tumor, beam filters are necessary for reducing contaminations of fast neutrons, thermal neutrons and gamma rays. For the thermal neutron beam BSA, Pb, and Bi were compared as gamma filter. As for the epithermal neutron beam BSA, Ni, and ^7^LiF were used as fast neutron filter and thermal neutron filter, respectively.

### Collimator

The collimator can limit divergence of the neutron beam and, reduce undesired irradiation and focus neutrons to patient position. And we calculated J/Φ to measure the beam divergence variation. A high ratio means that the neutron beam is close to the beam port and change slightly with distance from the port. A target value for this ratio should be > 0.7 (9).

### Clinical Parameters

Under clinical conditions, it is vital to investigate the dosimetry performance in the patients. So, in-phantom parameters were calculated. These parameters are advantage depth (AD), advantage ratio (AR), AD dose rate (ADDR), and treatment time (TT), where AD is the depth in phantom at which the total therapeutic dose in tumor equals the maximum dose of the normal tissue. AD indicates the depth of effective beam penetration. The AR is the ratio of the total therapeutic dose in tumor to the total normal tissue dose over a given depth (usually from the surface to AD). It is a measure of the therapeutic gain. ADDR is defined as the maximum dose rate for the normal tissue (15, 16).

Four components contributing to the absorbed dose in BNCT as follows:

1. The gamma dose Dγ: the dose due to gamma rays in the neutron beam as well as gamma rays induced in the tissue from nuclear reactions, like ^1^H (n,γ) ^2^H reaction;
2. The hydrogen dose D_H_: the dose due to recoil protons from ^1^H (n,n′) ^1^H reaction;
3. The nitrogen dose D_N_: the dose due to energetic proton and the recoiling ^14^C nucleus from ^14^N (n,p)^14^C reaction;
4. The boron dose D_B_: the dose due to α particles and the recoiling ^7^Li nuclei from ^10^B (n,α) ^7^Li reaction.

**Figure 3:**
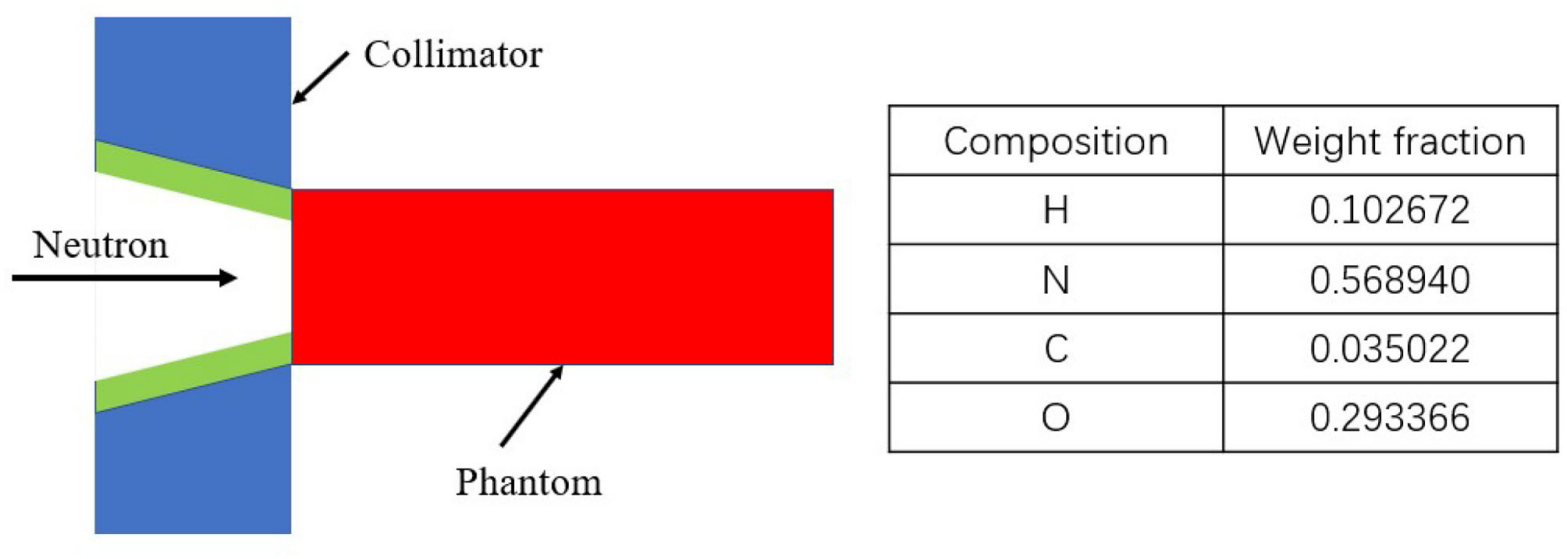
Geometry and composition of the phantom used in dose calculation.

The total RBE-weighted dose, D_T_ is expressed in the unit of RBE-Gy, as a sum of physical dose components multiplied by appropriate weighting-factors (RBE or CBE) for each dose component. It can be calculated using Equation (2) as below:

(2) $$\begin{array}{r} {\text{D}}_{\text{T}}={\text{C}}_{\text{B}}\times {\text{D}}_{\text{B}}+{\text{ω}}_{\text{N}}\times {\text{D}}_{\text{N}}+{\text{ω}}_{\text{H}}\times {\text{D}}_{\text{H}}+{\text{ω}}_{\gamma }\times {\text{D}}_{\gamma } \end{array}$$

where ω_γ_, ω_H_, ω_N_, and C_B_ are the weighting factors for gamma rays, hydrogen, nitrogen and boron, respectively. The values of ω_H_ and ω_N_ were taken as 3.0, ωγ was considered as 1, while ω_B_ was 1.35 for boron in the normal tissue and 3.8 for boron in the tumor. A simple phantom was considered to be a cylinder with a simplified composition of soft tissue as shown in Figure 3. The elemental compositions for the material of the cylinder phantom were also listed in Figure 3 (17). ^10^B was added to the phantom directly at the tumor concentration, as B_T_, of 30 ppm. The normal tissue concentration, B_N_, was chosen as 9 ppm, so that the ratio of B_T_ to B_N_, or T/N was 3.33 (18).

## Results and Discussion

### Optimization Design of the Thermal Neutron Beam BSA

#### Moderator for the Thermal Neutron Beam BSA

**Figure 4:**
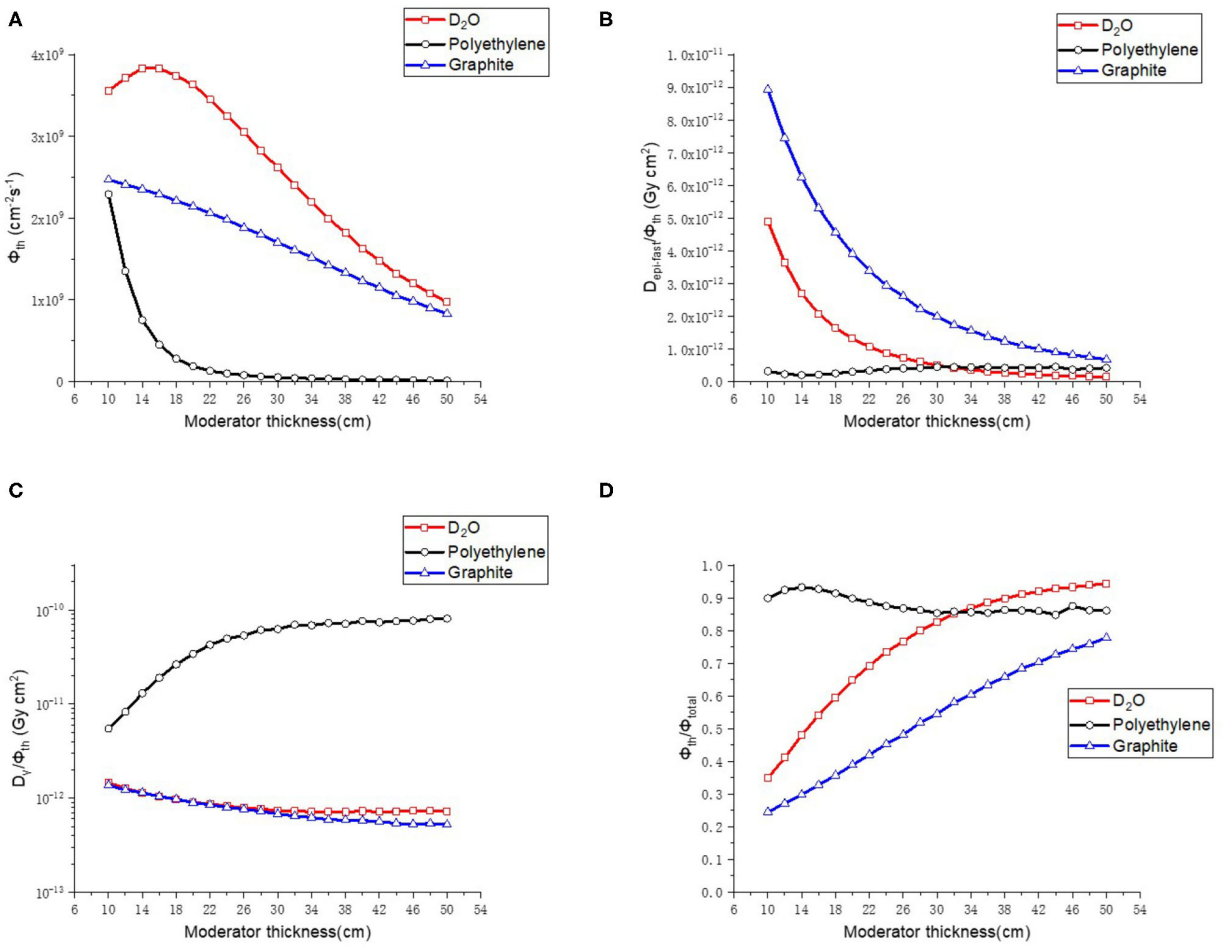
Thermal neutron parameters of moderator, **(A–D)** are thermal neutron flux, thermal neutron ratio, epithermal, and fast neutron component, Gamma component, respectively.

The thickness of the material varied from 10 to 50 cm with a step of 2 cm in the calculations, and the radius of the moderator was set to be 22 cm. The results are shown in Figure 4. D_2_O gave the highest thermal neutron flux (Φ_th_), and when the thickness exceeded 30 cm, it gave low epithermal and fast neutron component (D_epi−fast_/Φ_th_), low γ ray component (D_γ_/Φ_th_), and high proportion of thermal neutron (Φ_th_/Φ_total_). Therefore, D_2_O was chosen as the moderator for the thermal neutron beam BSA with a thickness of 40 cm.

#### Reflector for the Thermal Neutron Beam BSA

**Figure 5:**
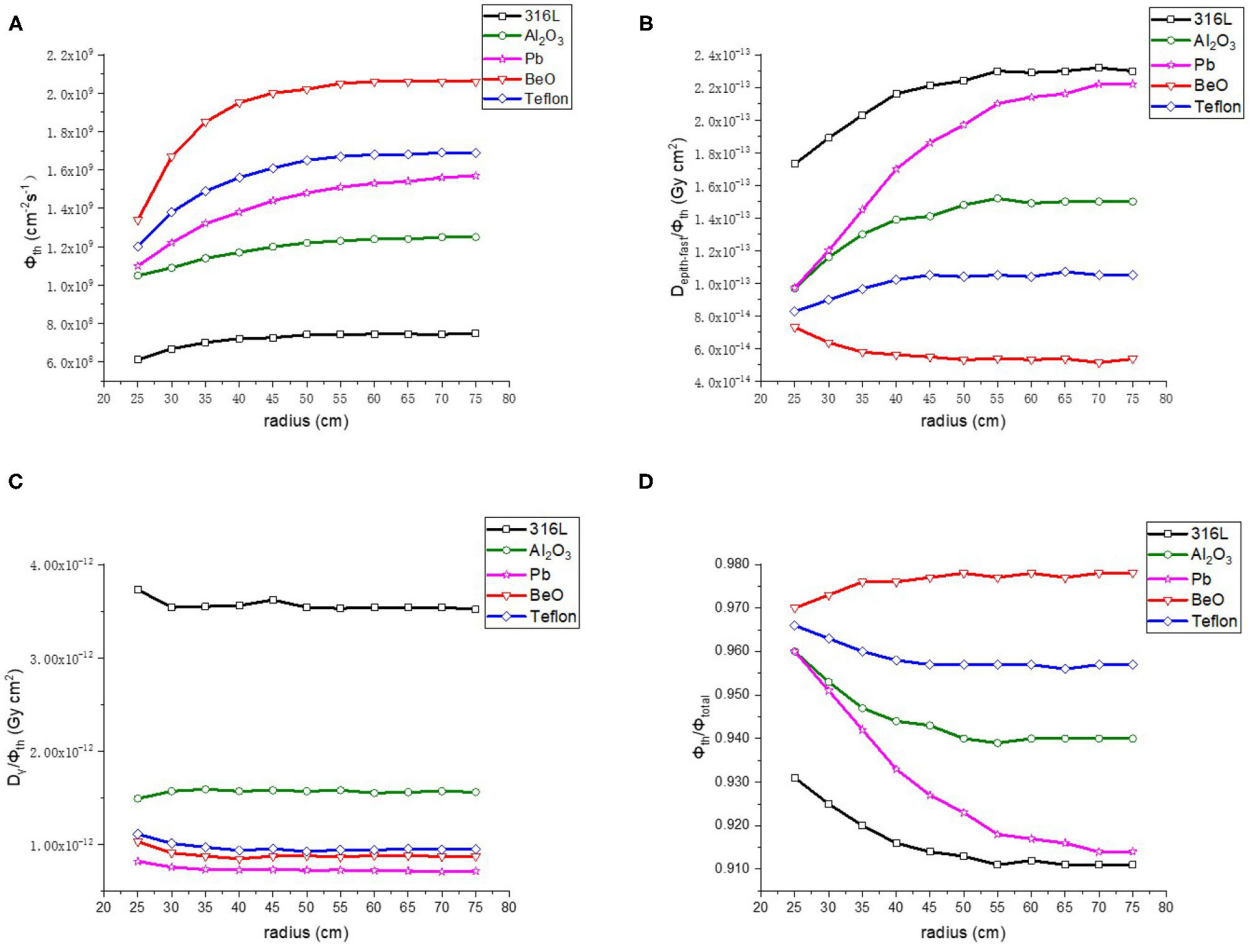
Thermal neutron parameters of reflector, **(A–D)** are thermal neutron flux, thermal neutron ratio, epithermal, and fast neutron component, Gamma component, respectively.

As the next step of the optimization, BeO, Al_2_O_3_, 316L stainless steel, Teflon and Pb were compared for the reflector. The radius of the reflector was changed with a range of 25–75 cm and a step length of 5 cm. The results are shown in Figure 5. First of all, among the five kinds of materials, BeO presented the highest thermal neutron flux (Φ_th_), the lowest epithermal and fast neutron component (D_epi−fast_/Φ_th_) and the highest proportion of thermal neutron (Φ_th_/Φ_total_). The γ ray component is also lower than 316L Stainless steel, Al_2_O_3_ and Teflon, so BeO was selected as the reflector material. In addition, it shows that when the radius is > 45 cm, the reflector radius has no significant influence on the neutron beam parameters. Considering the purpose of saving material and reducing the size of BSA, the reflector radius is chosen as 50 cm.

#### Gamma Filter for Thermal Neutron Beam BSA

**Figure 6:**
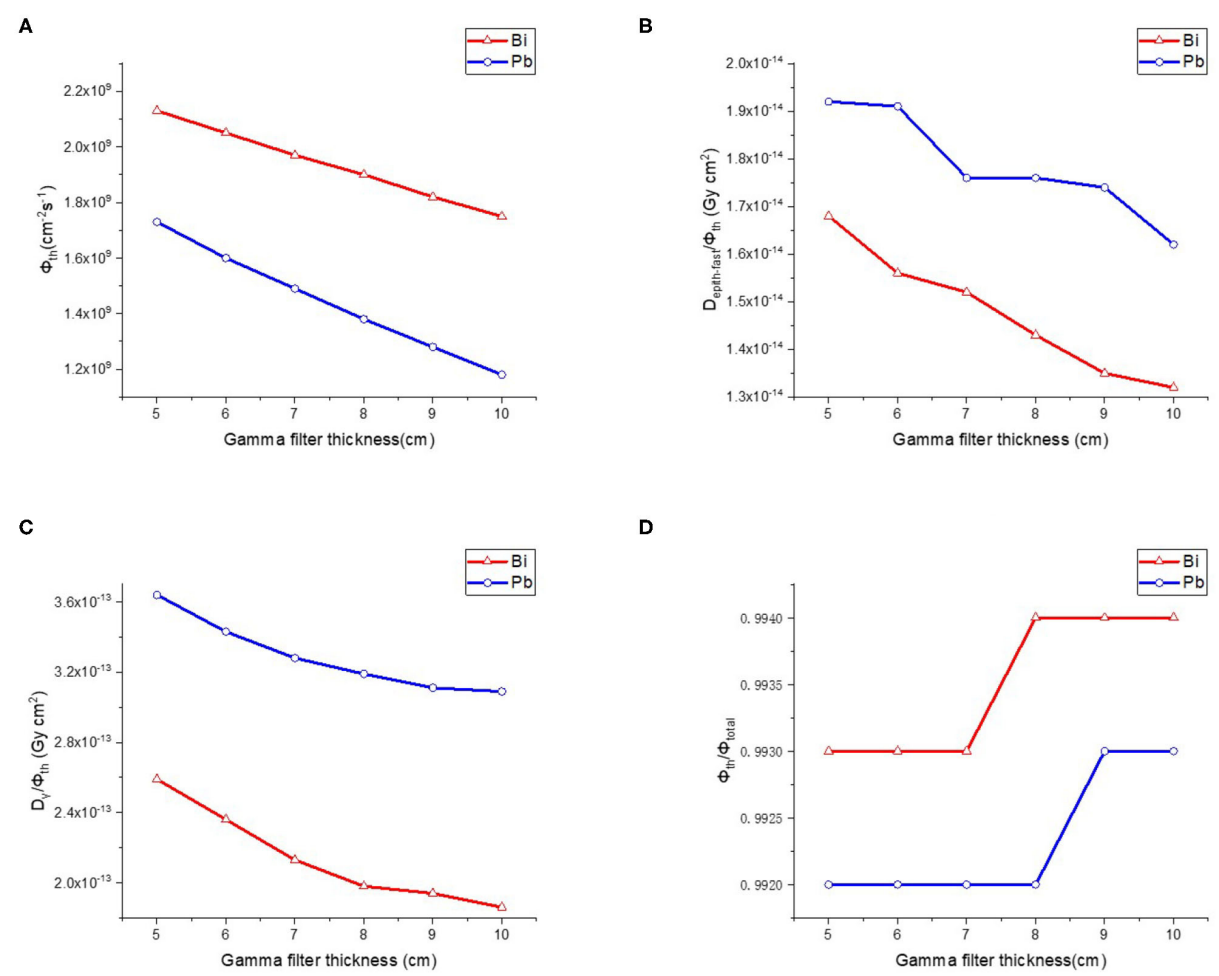
Thermal neutron parameters of gamma filter, **(A–D)** are thermal neutron flux, thermal neutron ratio, epithermal, and fast neutron component, Gamma component, respectively.

After determining the materials and sizes of the moderator and reflector, it is necessary to optimize the design of gamma filter to reduce γ rays' component because that γ rays which produced during moderation process cause unnecessary dose to normal tissue. The commonly used gamma filter materials are Pb and Bi. We made a comparison between these two materials. The thickness of gamma filter varied from 5–10 cm and the step length was 1 cm. Results as shown in Figure 6, Bi is a better choice for BNCT because it provides high photon elimination and low loss of thermal neutron flux. Therefore, we choose 9 cm Bi as gamma filter.

#### Collimator

**Figure 7:**
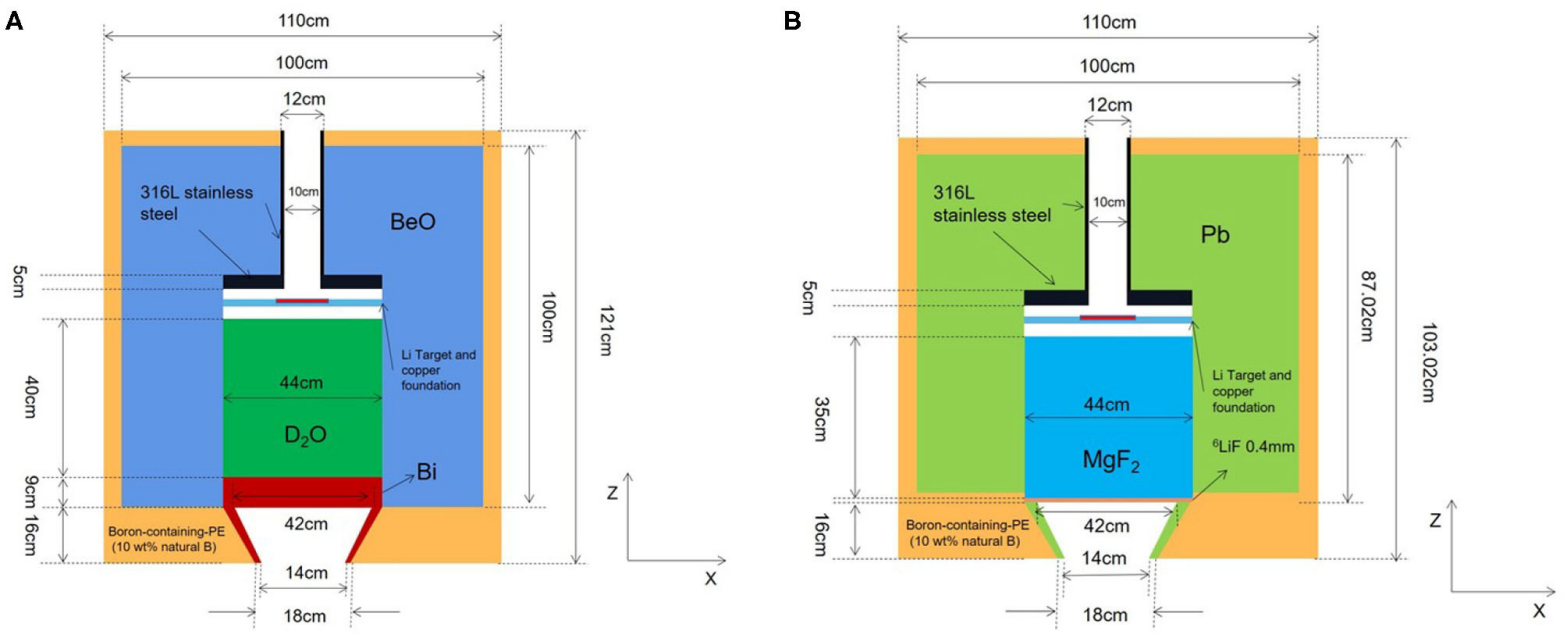
Structures of proposed epithermal and thermal neutron beam BSAs, **(A)** is thermal neutron BSA, and **(B)** is epithermal neutron BSA.

We choose Bi as material of collimator. And it is designed as a cone. It is 16 cm long (in height), and radius of bottoms are 22 and 7 cm. The J/Φ is 0.654 which is basically meets the target value. The details of collimator are shown in Figure 7A.

In conclusion, the final design of thermal neutron beam BSA can be obtained by using D_2_O as moderator, BeO as reflector, and Bi as gamma filter. The structure of thermal neutron beam BSA is shown in Figure 7A, and the neutron beam parameters were listed in Table 2 with other designs in the world. The flux of proposed thermal neutron beam BSA is higher than INFN and not much less than IHNI. However, IHNI is based on reactor, so it's difficult to build in hospital. So, the proposed thermal neutron beam BSA has its advantages.

**Table 2:** Thermal neutron beam parameters of proposed design and other designs in the world.

**Thermal neutron beam**	**Thermal neutron flux Φ_th_ (cm^–2^s^–1^)**	**Epithermal and fast neutron components D_epi-fast_/Φ_th_ (Gy cm^2^)**	**Thermal neutron ratio Φ_th_/Φ_total_**	**Gamma component D_γ_/Φ_th_ (Gy cm^2^)**	**J/Φ**
Proposed design	1.82 × 10^9^	1.35 × 10^−14^	0.994	1.94 × 10^−13^	0.654
Hospital neutron irradiator IHNI (12)	2.14 × 10^9^	1.70 × 10^−13^	–	9.73 × 10^−14^	0.798
Italian INFN (4 MeV protons on beryllium) (14)	1.17 ± 0.003 × 10^9^	8 ± 2 × 10^−16^	0.99	1.38 ± 0.003 × 10^−13^	–
The IAEA recommended values	≥1 × 10^9^	≤2 × 10^−13^	>0.9	≤2 × 10^−13^	>0.7

### Optimization Design of Epithermal Neutron Beam BSA

The process of the optimization design is basically the same as that of the thermal neutron beam BSA, so we show the final optimization results directly. We choose 45 cm MgF_2_ as moderator, 50 cm Pb as reflector, 0.4 mm ^6^LiF as the thermal neutron filter. The collimator is as same as thermal neutron BSA except its material is Pb. The structure of epithermal neutron beam BSA is shown in Figure 7B, and the parameters are listed in Table 3 with other designs in the world.

**Table 3:** Epithermal neutron beam parameters of proposed design and other designs in the world.

**Epithermal neutron beam**	**Epithermal flux Φ_epith_ (cm^–2^s^–1^)**	**Fast neutron component D_fast_/Φ_epith_ (Gy cm^2^)**	**Thermal neutron ratio Φ_th_/Φ_epith_**	**Gamma component D_γ_/Φ_th_ (Gy cm^2^)**	**J/Φ**
Proposed design	1.26 × 10^9^	1.85 × 10^−13^	0.033	1.48 × 10^−13^	0.715
Kyoto University (8)	1.2 × 10^9^	5.8 × 10^−13^	–	7.8 × 10^−14^	–
Nagoya University (7)	1.05 × 10^9^	2 × 10^−13^	0.058	2.19 × 10^−13^	0.71
Montagnini et al. (19)	1.226 × 10^9^	1.7 × 10^−13^	0.0096	1.7 × 10^−13^	0.61
Kim et al. (20)	1.01 × 10^9^	0.09 × 10^−13^	0.048	0.09 × 10^−13^	–
Kim et al. (20)	1.03 × 10^9^	0.08 × 10^−13^	0.047	0.08 × 10^−13^	–
Fantidis. (21)	1.096 × 10^9^	1.40 × 10^−13^	0.0056	1.40 × 10^−13^	–
Fantidis. (21)	0.523 × 10^9^	1.77 × 10^−13^	0.0098	1.77 × 10^−13^	–
The IAEA recommended values	≥1 × 10^9^	≤2 × 10^−13^	<0.05	≤2 × 10^−13^	>0.7

It is obvious that all the parameters of proposed BSAs fulfill the IAEA recommended values. And the flux of proposed epithermal neutron beam BSA is higher than other designs.

### Calculation of Clinical Parameters

**Figure 8:**
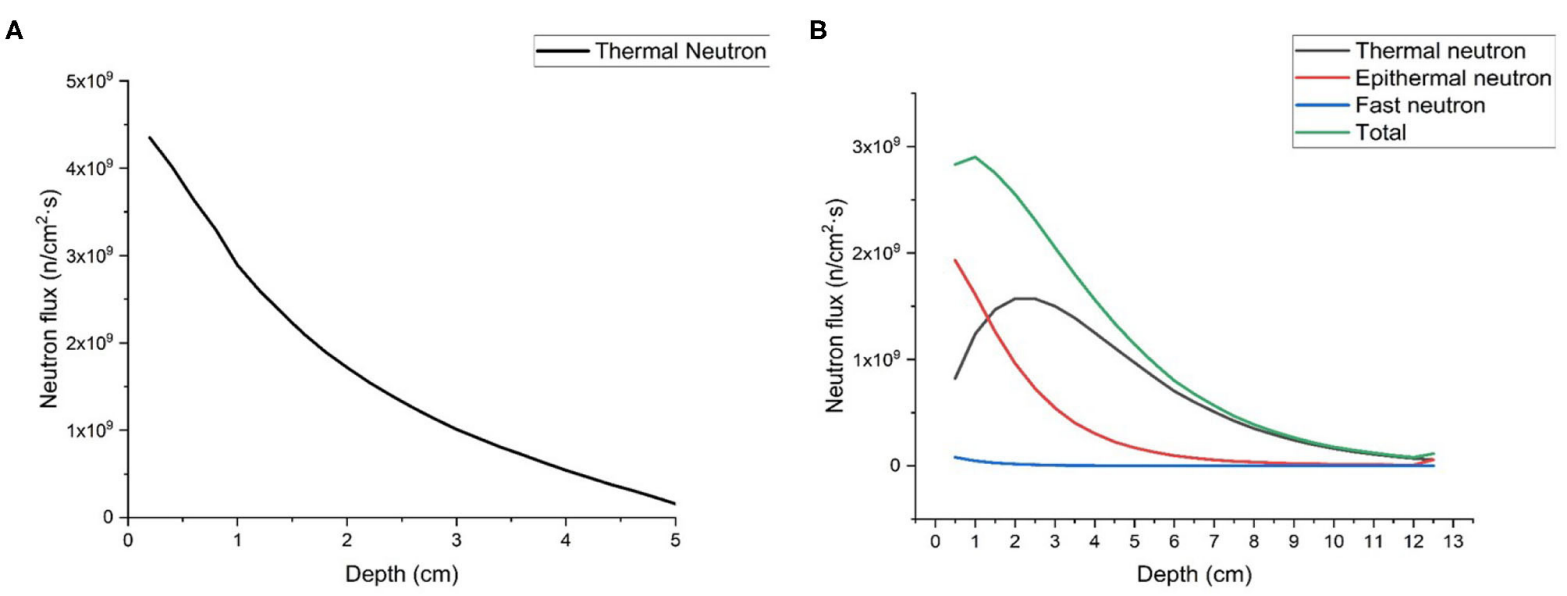
Neutron flux distribution in the phantom, **(A)** is thermal neutron beam, and **(B)** is epithermal neutron beam.

Firstly, we calculated neutron flux distribution in the phantom of thermal neutron beam and epithermal neutron beam generated by proposed BSAs, respectively. As shown in Figures 8A,B, the maximum depth of flux in the phantom of thermal neuron beam is 5 cm, and the maximum depths of thermal and epithermal flux of epithermal neutron beam are 12 and 8 cm, respectively. The components of epithermal and fast neutron are too low so that they are not shown in the Figure 8A.

**Figure 9:**
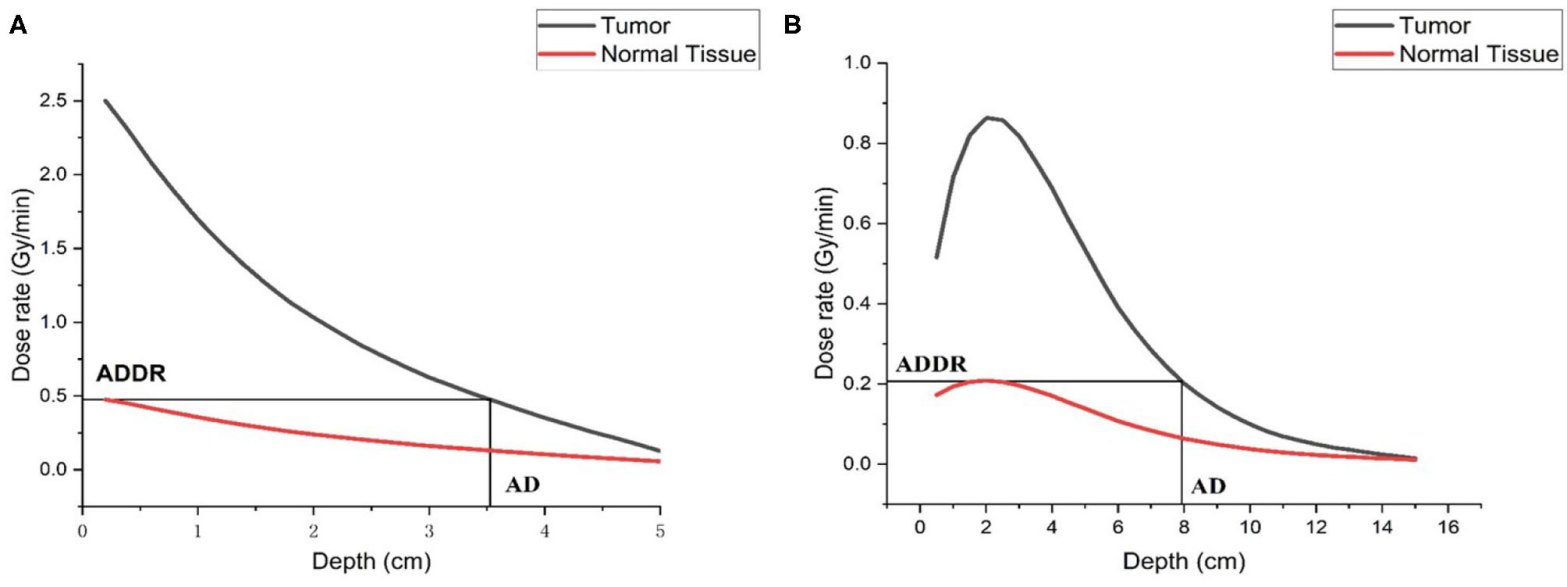
Dose distribution in the phantom, **(A)** is thermal neutron beam, and **(B)** is epithermal neutron beam.

As for the dose in tumor and normal tissue, we also calculated them in the phantom. As shown in Figure 9, the AD is 7.93 cm for epithermal neutron beam and 3.52 cm for thermal neutron beam. The ADDR for thermal neutron beam is 0.476RBE-Gy/min, and for epithermal neutron beam it is 0.208RBE-Gy/min. According to the results, we found that the large amount of dose was delivered to skin and superficial normal tissue. If we defined the time that dose delivered to normal tissue exceed the maximum tolerated dose (12.5RBE-Gy) as the treatment time (TT) which is the maximum value. Then, TTs are 60.1 min for epithermal neutron beam and 26.3 min for thermal neutron beam. During this time, the maximum D_T_s of tumor are 51.87 RBE-Gy for epithermal neutron beam and 65.75 RBE-Gy for epithermal neutron beam. And maximum DRs are 0.863RBE-Gy/min for epithermal neutron beam and 2.5 RBE-Gy/min for thermal neutron beam, respectively.

## Conclusions

In this paper, MCNPX is used to design the BSAs of an AB-BNCT using ^7^Li(p,n)^7^Be reaction. The optimized BSAs for thermal and epithermal neutron beams can ensure that the beam parameters at the exit all meet the recommended values of IAEA. And the clinical parameters are also calculated so that it can give a reference for clinical condition. So the final optimal design of thermal and epithermal BSA can be an important reference for the BSA engineering scheme of multi-terminal AB-BNCT device. In the follow-up work, the accelerator-based multi-terminal BSA conversion device will be further designed to facilitate the flexible switching and replacement of BSA, ensure the safe operation of the device, and give better play to the advantages of multi-terminal devices, which will promote the development of AB-BNCT in China.

## Data Availability Statement

The raw data supporting the conclusions of this article will be made available by the authors, without undue reservation.

## Author Contributions

GL adapted the neutron source in the Li target (with WJ), performed the simulations on BSA, calculated clinical parameters, and wrote the manuscript. WJ and LZ helped execute MCNP and adjust the input files. WC participated in the study design and reviewed the results. QL supervised this work and contributed to the study design. All authors contributed to the article and approved the submitted version.

## Conflict of Interest

The authors declare that the research was conducted in the absence of any commercial or financial relationships that could be construed as a potential conflict of interest.
